# Pro-Asthmatic Cytokines Regulate Unliganded and Ligand-Dependent Glucocorticoid Receptor Signaling in Airway Smooth Muscle

**DOI:** 10.1371/journal.pone.0060452

**Published:** 2013-04-04

**Authors:** Aihua Hu, Maureen B. Josephson, Barry L. Diener, Gustavo Nino, Shuyun Xu, Chinmay Paranjape, Jordan S. Orange, Michael M. Grunstein

**Affiliations:** 1 Division of Pulmonary Medicine, Children's Hospital of Philadelphia Research Institute, University of Pennsylvania Perlman School of Medicine, Philadelphia, Pennsylvania, United States of America; 2 Department of Integrative Systems Biology and Division of Pulmonary & Sleep Medicine, Children's National Medical Center, Center for Genetic Medicine Research, George Washington University, Washington, D.C., United States of America; 3 Section of Immunology, Allergy and Rheumatology, Texas Children's Hospital, Baylor College of Medicine, Houston, Texas, United States of America; University of Padua, Italy

## Abstract

To elucidate the regulation of glucocorticoid receptor (GR) signaling under pro-asthmatic conditions, cultured human airway smooth muscle (HASM) cells were treated with proinflammatory cytokines or GR ligands alone and in combination, and then examined for induced changes in ligand-dependent and -independent GR activation and downstream signaling events. Ligand stimulation with either cortisone or dexamethsone (DEX) acutely elicited GR translocation to the nucleus and, comparably, ligand-independent stimulation either with the Th2 cytokine, IL-13, or the pleiotropic cytokine combination, IL-1β/TNFα, also acutely evoked GR translocation. The latter response was potentiated by combined exposure of cells to GR ligand and cytokine. Similarly, treatment with either DEX or IL-13 alone induced GR phosphorylation at its serine-211 residue (GR^Ser211^), denoting its activated state, and combined treatment with DEX+IL-13 elicited heightened and sustained GR^Ser211^ phosphorylation. Interestingly, the above ligand-independent GR responses to IL-13 alone were not associated with downstream GR binding to its consensus DNA sequence or GR transactivation, whereas both DEX-induced GR:DNA binding and transcriptional activity were significantly heightened in the presence of IL-13, coupled to increased recruitment of the transcriptional co-factor, MED14. The stimulated GR signaling responses to DEX were prevented in IL-13-exposed cells wherein GR^Ser211^ phosphorylation was suppressed either by transfection with specific serine phosphorylation-deficient mutant GRs or treatment with inhibitors of the MAPKs, ERK1/2 and JNK. Collectively, these novel data highlight a heretofore-unidentified homeostatic mechanism in HASM cells that involves pro-asthmatic cytokine-driven, MAPK-mediated, non-ligand-dependent GR activation that confers heightened glucocorticoid ligand-stimulated GR signaling. These findings raise the consideration that perturbations in this homeostatic cytokine-driven GR signaling mechanism may be responsible, at least in part, for the insensirtivity to glucocorticoid therapy that is commonly seen in individuals with severe asthma.

## Introduction

Due to their potent immunosuppressive and anti-inflammatory properties, glucocorticoids (GCs) are commonly used as the primary treatment for many autoimmune, proinflammatory and allergic diseases. GCs act by binding to the intracellular glucocorticoid receptor (GR), thereby enabling it to dissociate from its chaperone proteins, become phosphorylated, and translocate as a homodimer to the nucleus. The intra-nuclear ligand-activated GR can then initiate gene transactivation by directly interacting with specific DNA sequences, the glucocorticoid response elements (GREs), residing in the promotor region of a large array of GC-responsive genes. Moreover, the GR can inhibit gene expression via transrepression that involves binding to negative GRE sequences or direct binding of the GR to specific transcription factors such as AP-1 and NF-κB, thereby inhibiting their potential to stimulate proinflammatory gene expression [1]. The latter mechanism is believed to be importantly involved in mediating the anti-inflammatory actions of GCs [2].

Tissue responsiveness to GCs under proinflammatory conditions may be modulated by changes in the concentration of specific cytokines and other extracellular molecules in the tissue's microenvironment [3]. Under these circumstances, altered GC responsiveness may be attributed to induced changes in the tissue's ability to regulate its own bioavailability of GC and/or changes in its GR signaling mechanism. Regarding the former mechanism, it has been demonstrated in different cell types that various cytokines can evoke upregulated expression of the endogenous GC-activating enzyme, 11ß-hydroxysteroid dehydrogenase type 1 (11ß-HSD1), that converts the inactive 11β-oxoglucocorticoid, cortisone, to its bioactive derivative, cortisol [4]–[7]. This suggests that the localized adverse effects of specific proinflammatory cytokines may be mitigated by their induced upregulation of 11ß-HSD1 oxoreductase activity, the latter phenomenon serving an anti-inflammatory feedback function by facilitating endogenous GC bioavailability at the affected tissue site. Comparably, GR function can also be modulated under proinflammatory conditions, as demonstrated by increased GR expression following exposure of different cell types to proinflammatory cytokines [8]–[11], which may confer heighten GC responsiveness. Moreover, the GR is subject to post-translational modifications that alter its state of activation and signaling and, conbsequently, the net response to glucocorticoid exposure.

As a phosphoprotein that is structurally similar to other members of the superfamily of nuclear receptors, the human GR is subject to phosphorylation at several serine sites that are clustered in the N-terminal region containing the transcriptional activation function-1 (AF1) domain [12], [13]. The kinases that phosphorylate human GR principally include MAPKs, cyclin-dependent kinases, and glycogen synthase kinase-3 [12]–[16]. While hormone binding coupled to GR phosphorylation is critical for mediating its function, recent evidence demonstrates that the GR is also susceptible to phosphorylation in the absence of ligand, and GR hyperphosphorylation can occur when hormone is present [12], [13]. When hyperphosphorylated, the ligand-activated GR exhibits heightened binding to DNA and, in association with increased co-factor recruitment, targeted GC-responsive gene expression is enhanced [13], [17]. More recently, significant insight has been gained into the mechanisms by which GR phosphorylation regulates gene transcriptional activity and repression, with evidence demonstrating that the cluster of phosphorylation sites in the AF1 domain serve different functions. In the human GR, these include the serine sites, Ser^203^, Ser^211^ and Ser^226^ that are also conserved among other species and, largely based on studies involving mutations at these sites, their relative levels of phosphorylation were shown to regulate GR localization, co-factor recruitment, and gene-specific transactivation or transrepression [18]. Accordingly, whereas Ser^203^ phosphorylation has been associated with nuclear translocation of GR, intra-nuclear Ser^211^ phosphorylation was implicated in facilitating GR interaction with the transcriptional co-factor, MED14, and, hence, gene-specific GR transactivation, while Ser^226^ phosphorylation was associated with GR transrepression and enhanced GR nuclear export [18]–[20].

In light of the above evidence, and given the important role that GCs play in suppressing the inflammatory response and altered airway contractility in asthma [1], this study examined the regulation of GR signaling in proinflammatory cytokine-stimulated human airway smooth muscle (HASM) cells. The results are the first to identify that: 1) HASM cells exposed to the key pro-asthmatic Th2-type cytokine, IL-13, exhibit ligand-independent GR activation associated with nuclear translocation coupled to site-specific GR phosphorylation; and 2) these cytokine-induced ligand-independent effects confer increased GC hormone-stimulated GR transcriptional activity, thereby serving to homeostatically enhance GC responsiveness. To the extent that this new evidence suggests that proinflammatory cytokine-induced ligand-independent GR signaling may importantly contribute to heightening GC sensitivity in asthma, future interventions targeted at this homeostatic signaling mechanism may lead to new approaches to enhance the efficacy of GC therapy.

## Methods

### Materials

All chemicals were purchased from Sigma-Aldrich unless otherwise indicated. The human airway smooth muscle (HASM) cells were obtained from Bio Whittaker, Inc.

### Culture and treatment of ASM cells

HASM cells were grown in SmBm media supplemented with 10% FBS (Bio Whittaker Inc.) and maintained throughout in a humidified incubator containing 5% CO2 in air at 37°C. The experimental protocols involved growing the cells to near confluence in the above medium. Thereafter, in separate studies, the cells were starved in unsupplemented Ham's F12 media for 24 hr, and then examined under different experimental conditions described below.

### Assessment of GR translocation

ASM cells were grown on 4-chamber slides to ∼85% confluence. The cells were treated with 100 nM cortisone or dexamethasone (DEX) for 30 min alone or in combination, both in the absence and presence of co-treatment with either vehicle or maximally effective concentrations of either IL-13 (50 ng/ml) or the combination of IL-1β and TNF-α (10 and 100 ng/ml, respectively [21], [22]. Cells were also treated with either of these cytokines in absence of hormone exposure. Following treatment, the cells were fixed with 3.7% formalin to stop the reaction and then stained for GR using a primary mouse anti-human GRα antibody and a FITC-conjugated goat-anti-mouse IgG secondary antibody. Cells were rinsed and then mounted in Vectashield mounting media with DAPI (Vector Laboratories). An Olympus IX-81 DSU spinning disk confocal microscope was used to acquire the images at 60× magnification using a 1.49 NA objective. 10–15 cells for each condition were selected for staining clarity, focal plane uniformity and shape. The majority of the analysis was derived from images of the cell bodies. Cells were analyzed using Improvision Volocity classification software (Improvision/Perkin Elmer), DAPI and FITC area and intensity were measured. Ratio of intensity of FITC (GR) in the DAPI-stained nuclear area to that found over the entire cell was calculated. Individual comparative two-tailed t-tests were conducted to compare the control and treatment samples.

### Transfection of HASM cells with Ser*^203^* and Ser*^211^* phosphorylation-deficient mutant GRs

HASM cells were transfected to over-express either hemagglutinin (HA)-tagged CMV promotor-driven wild-type (WT) human GR (pCMV-HA-hGR) or the alanine-substituted for serine phosphorylation-deficient site-specific mutant GRs, pCMV-HA-hGRS203A (S203A) or pCMV-HA-hGRS211A (S211A) (plasmids kindly provided by M. Garabedian; NYU, New York, NY). The transfections are performed using a modified previously described method [14], [25]. Briefly, HASM cells were seeded into 6-well plates and, at ∼40% confluency, the medium was replaced with the reduced serum-containing medium, Opti-MEM (Invitrogen). The cells were then transfected with WT or either of the serine site-specific mutant GR, using Oligofectamine (Invitrogen) as the transfection agent. Efficiency of transfection was then assessed by indirect immunofluorescence in cells co-expressing vector-encoded GFP, and immunoblotting with HA- and GR-specific antibodies. The efficacy of transfection ranged between 64 and 76%.

### Western blot analyses

Confluent HASM cell cultures were serum-starved for 24 hr and then stimulated with IL-13 or DEX alone and in combination for varying durations. Cytoplasmic and nuclear fractions of the cells were then isolated using the NucBuster Protein Extraction Kit (EMD Biosciences). Equal amounts of proteins (25 µg) were separated by SDS-PAGE and the extracts were treated with antibodies at 1∶1000 dilution directed against total GRα (BD Biosciences) and the Ser^203^- and Ser^211^-specific phosphorylated forms of GR (Abcam abd Cell Signaling Technology, respectively). In comparable experiments, expression of the latter phosphorylated forms of GR was also examined in HASM cells that were transfected with the above WT and mutant S203A and S211A constructs. Finally, total GR and β-actin protein levels were detected in WT- and mutant GR-transfected control and IL-13-treated cells. HRP-conjugated antibody against rabbit IgG was used as a loading control. The reaction was detected using enhanced chemiluminescence, and protein band intensities were quantified by densitometry.

### EMSA analysis of GR:DNA binding

HASM cells were starved overnight and then treated for varying durations with either IL-13 or DEX. As previously described [24], cell suspensions were then prepared after trypsinization, and nuclear protein was extracted using the NucBuster Protein Extraction kit (Novagen). Gel-shift assay was performed using Panomics EMSA kits that involved incubating the biotin-labeled GR transcription factor probe or excess unlabelled probe with the nuclear protein extracts. The mixture was then separated on non-denaturing polyacrylamide gels and the shifted bands corresponding to the protein/DNA complexes were visualized after exposure to X-ray film.

### Quantitative assessment of GR:DNA binding activity

Nuclear extracts were isolated from control HASM cells and cells treated for 60 min with varying concentrations of DEX, both in the absence and presence of pretreatment for 24 hr with either vehicle alone or IL-13. A sensitive ELISA-based transcription factor binding assay kit (TransAM) was then used to quantitatively assess the binding activity of GRα to its consensus DNA sequence in the nuclear extracts. The protein concentration of each sample was determined by BCA. Extracts were were incubated in 96-well plates coated with GR binding consensus oligonucleotide sequence for 1 h, then incubated with the supplied primary anti-GR antibody for 1 h, and with a peroxidase-conjugated secondary antibody for 1 h. After the substrate was added, color development was read at 450 nm, and the OD of GR was recorded.

### GRE-driven transactivation

GR-dependent transcriptional activation was assayed using the Pathway Profiling System from Clontech (Mountain View, CA), as previously described [25]. Briefly, after grown to ∼85% confluence, HASM cells were transfected using calcium phosphate with a vector containing a promoter-reporter construct, pGRE-SEAP, that is comprised of three glucocorticoid response element (GRE) sequences upstream from a TATA box and a reporter gene that encodes a secretable form of alkaline phosphatase (SEAP). Cells were also transfected with pTAL-SEAP, an identical plasmid but without the GRE promotor, serving as a negative control, as well as a plasmid expressing β-galactosidase, (pCMVSport-β-gal) serving as an internal control. After 18 hours of transfection, the medium was changed to fresh serum-free Ham's F-12 media for 8 hours. Cells were then treated for 24 hr either with DEX (1 µM) or IL-13 (50 ng/ml) under different experimental conditions, as described. Thereafter, the conditioned culture medium was harvested and assayed for SEAP activity using the Great EscAPe SEAP chemiluminescence kit from Clontech, according to the manufacturer's protocol.. The measured levels of AP activity were then normalized to β-galactosidase activity in order to correct for any variations caused by differences in transfection efficiency between the cell culture wells.

### Co-immunoprecipitation

Co-immunoprecipitation assays were performed under native conditions in order to preserve protein–protein associations. As previously described [26], after indicated treatment, cells were harvested and then lysed with lysis buffer. 1.5 mg of Protein G Dynabeads (Invitrogen) were incubated with 10 µg of anti-GR rabbit IgG (Millipore) and incubated for 10 min at room temperature with rotation. Following several washes, the bound bead/antibody complex was added to sample, mixed by pipetting, and incubated for 2 h at 4°C with rotation. The captured bead/Protein G/antigen complex was washed several times and eluted at pH 3.0 with rotation at room temperature. The precipitated immunocomplexes were analyzed by immunoblotting with anti phospho-GR^Ser211^ and MED14 antibody.

### Statistical analyses

Results are presented as means ± SE or ± SD values, where indicated. Comparisons between groups were performed using the unpaired Student's t-test or one-way ANOVA, with Tukey-Kramer post hoc testing, where appropriate. A probability of <0.05 was considered statistically significant. Statistical analyses were conducted using the Prism computer program by GraphPad Software Inc.

## Results

### Cytokine regulation of ligand-dependent and -independent GR nuclear translocation in HASM cells

**Figure 1:**
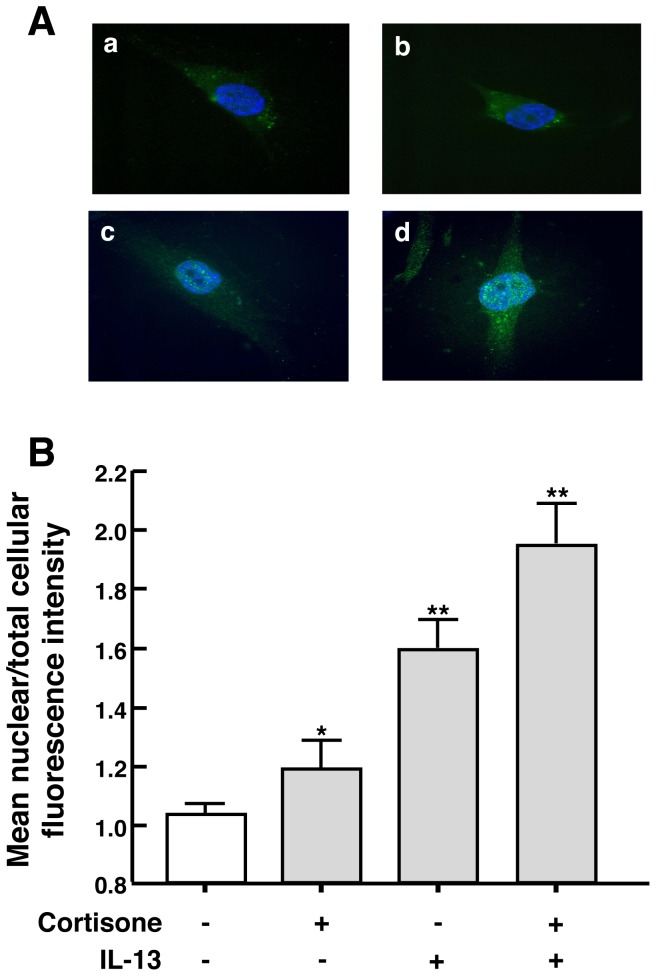
Separate and cooperative effects of DEX-stimulated and unliganded IL-13-induced nuclear translocation of GR in HASM cells. GR localization detected by Immunofluorescence staining (A) and its quantification (B) depicting that, relative to diffuse cytoplasmic distribution of GR in vehicle-exposed (control) HASM cells (*a*), treatment for 30 min with either cortisone (*b*) or IL-13 alone (*c*) elicited increased intra-nuclear localization of GR. which was further enhanced in cells co-treated with cortisone ± IL-13 (*d*). Note: Induced nuclear translocation of GR by IL-13 alone denotes the presence of ligand-independent GR activation, and heightened GR translocation exhibited by cells treated with cortisone ± IL-13 demonstrates cooperative effects of ligand (cortisone)-induced and IL-13-induced ligand-independent GR activation. Data represent mean±SD values based on an average of 25 HASM cells examined over 3–5 repeats (*p<0.05; **p<0.01).

To determine the role of proinflammatory cytokines in regulating GR signaling in HASM cells, we initially examined the effects of cytokine exposure on nuclear translocation of the stimulated GR, a critical feature of GR activation. Confluent HASM cell cultures were exposed for 30 min to either the endogenous GC, cortisone (100 nM) or the synthetic GC, dexamethasone (DEX; 100 nM), both in the absence and presence of co-treatment with a maximally effective concentration of either IL-13 (50 ng/ml) or the combination of IL-1β and TNF-α (10 and 100 ng/ml, respectively). The representative photomicrographs in Fig. 1A depict immunofluorescence staining of GR detected in cells exposed to either vehicle, cortisone or IL-13 alone, and cortisone+IL-13; and Fig. 1B displays the corresponding quantitative analysis of the effects of these treatment conditions on nuclear-to-total cellular fluorescence intensity based on repeated measurements made in DAPI-stained cells. Relative to the diffuse cytoplasmic distribution of GR detected in vehicle-exposed (control) cells (*a*), cortisone-treated cells exhibited increased intra-nuclear localization of GR (*b*); and further markedly enhanced intra-nuclear GR localization was detected in cortisone-exposed cells that were co-treated with IL-13 (*d*). Interestingly, distinctly increased intra-nuclear localization of GR was also exhibited in cells that were treated for 30 min with IL-13 alone (*c*), the latter observation demonstrating the presence of acute cytokine-induced ligand-independent nuclear translocation of GR in HASM cells. Qualitatively, similar induction of hormone-dependent and -independent nuclear translocation of GR was also detected in HASM cells that were comparably exposed to either DEX or IL-1β/TNF-α alone and in combination (Fig. S1). Together, these observations demonstrate that HASM cells display both GC ligand-dependent and cytokine-induced ligand-independent nuclear translocation of GR, and that these phenomena act cooperatively whether elicited either by a synthetic or endogenous GC, and either the Th2 cytokine, IL-13, or the pleiotropic proinflammatory cytokines, IL-1β and TNFα.

### Cytokine regulation of ligand-dependent and -independent GR phosphorylation and DNA binding activity

**Figure 2:**
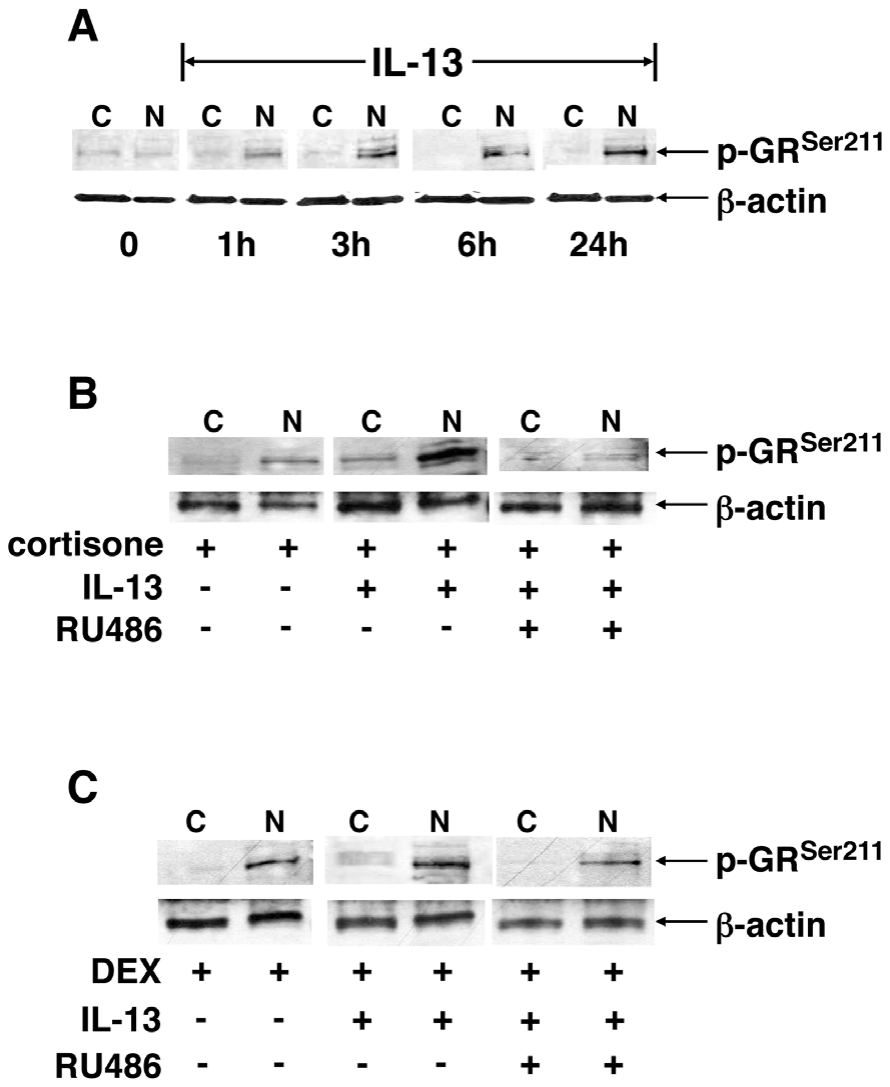
Immunoblot analysis of separate and combined effects of ligand-stimulated and unliganded IL-13-induced GR^Ser211^ phosphorylation in HASM cells. (**A**) Treatment with IL-13 elicits progressive increases in p-GR^Ser211^ for up to 24 h following cytokine exposure that are detected in the nuclear (N) and not the cytoplasmic (C) fraction. Induction of p-GR^Ser211^ by cortisone (**B**) or DEX (**C**) is increased in HASM cells initially exposed for 24 h to IL-13; and the p-GR^Ser211^ response is markedly suppressed in cells pretreated with the GR antagonist, RU486.

IL-13-induced activation of the unliganded GR was further substantiated when examining the effects of the cytokine on GR phosphorylation at its Ser^211^ residue, which denotes the activated state of the GR that is largely confined to the nucleus and is associated with transactivation of GC-responsive genes [16], [18], [23]. Immunoblot analysis using a mAb that specifically detects Ser^211^-phosphorylated GR (p-GR^Ser211^) demonstrated that HASM cells treated for 1–24 h with IL-13 (50 ng/ml) alone exhibited temporal increases in p-GR^Ser211^ that were evidenced in the nuclear (N) but not in the cytoplasmic (C) fraction, with sustained increased phosphorylation detected at 24 h (Fig. 2A). Moreover, relative to cells exposed for 60 min to 100 nM of either cortisone or DEX alone (Figs. 2B and 2C, respectively), distinctly increased p-GR^Ser211^ was detected in both cortisone- and DEX-exposed cells that were pretreated with IL-13 (50 ng/ml×24 h), and this enhanced GR phosphorylation was suppressed in cells that were co-treated with the GR antagonist, RU486 (10 µM). Collectively, these data demonstrate that, independent of endogenous or synthetic GC binding to GR, IL-13 elicits nuclear translocation of the unliganded Ser^211^-phosphorylated GR and that, in the presence of either endogenous or synthetic GC ligand, IL-13 acts cooperatively with the ligand to further enhance intra-nuclear p-GR^Ser211^ accumulation.

**Figure 3:**
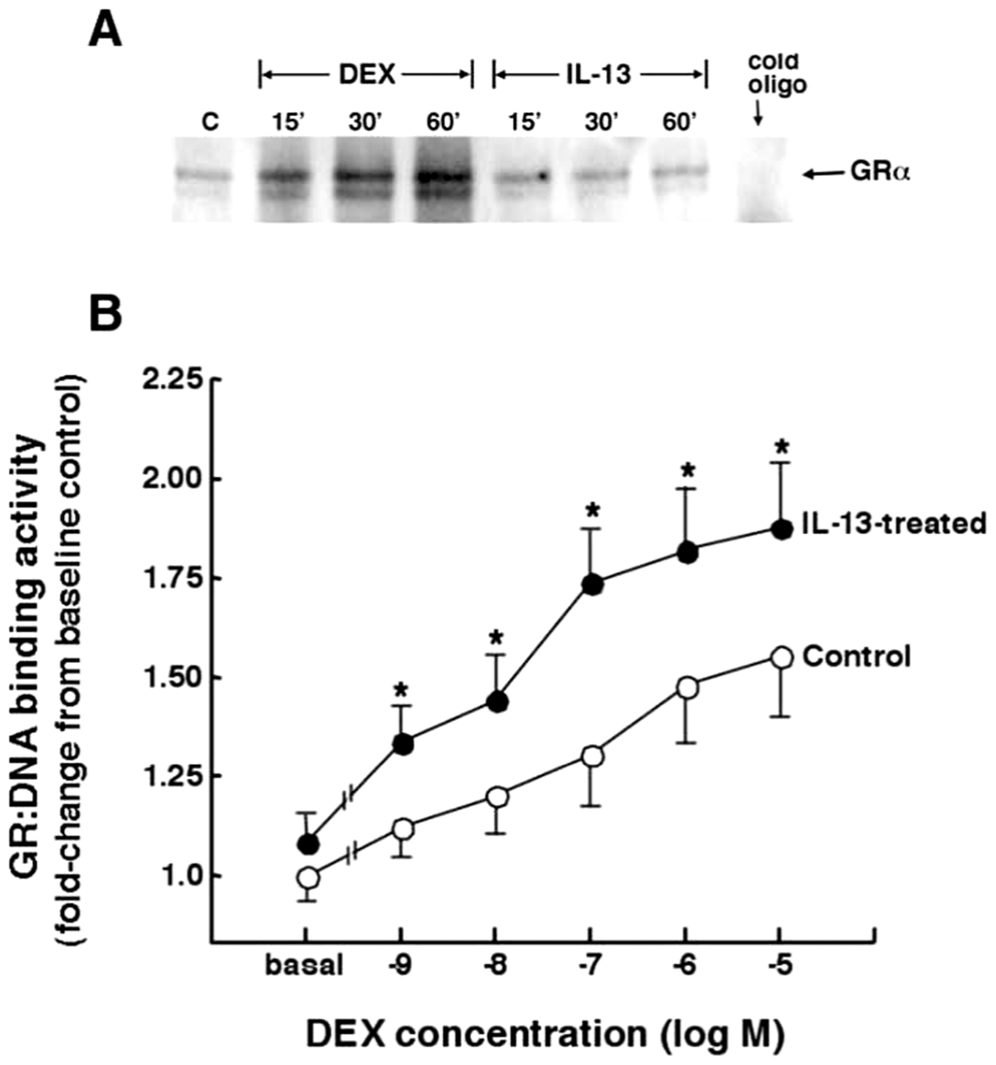
Comparison of DNA binding activity of GR in HASM cells exposed to either DEX or IL-13 alone and in combination. (**A**) EMSA depicting that GR binding to its consensus DNA sequence is acutely elicited by ligand stimulation with DEX, whereas treatment with IL-13 alone does not evoke GR:DNA binding. (**B**) Following 24 h exposure to vehicle alone (control), HASM cells treated with DEX exhibit dose-dependent stimulation of GR:DNA binding activity, the latter quantified using a sensitive ELISA kit. GR:DNA binding activity is significantky increased at all administered doses of DEX in cells pretreated for 24 h with IL-13. Data are mean ± SE values based on 4–5 determinations at each administered dose of DEX. *p<0.05.

We next investigated whether the cytokine-induced increase in intra-nuclear bioavailability of the activated GR confers heightened ligand-independent and/or hormone-stimulated downstream GR signaling. As shown in Fig. 3A, using EMSA to detect GR binding to its consensus DNA sequence (GRE), contrasting the expected acute progressive induction by DEX (1 µM) of GR binding to DNA over 60 min, HASM cells treated with IL-13 alone (50 ng/ml) did not exhibit induced GR:DNA binding activity. By comparison, as shown in Fig. 3B, when evaluating whether pretreatment with IL-13 alters DEX-stimulated GR:DNA binding activity, the latter quantitatively assessed using a highly sensitive ELISA assay, relative to control (vehicle-treated) cells, dose-dependent stimulation of GR:DNA binding activity by DEX was significantly increased in HASM cells that were pretreated with IL-13 (50 ng/ml×24 h). Taken together, these results demonstrate that, although IL-13 can independently evoke translocation and intra-nuclear accumulation of p-GR^Ser211^, this unliganded “early” GR activation by IL-13 does not confer GR interaction with DNA but, rather, the response to IL-13 serves to heighten HASM sensitivity to hormone-stimulateed GR:DNA binding activity. In light of this evidence, a series of studies were pursued to elucidate the potential mechanism underlying IL-13-induced enhanced GC ligand-dependent GR activation, and determine whether this mechanism enables heightened GR transactivation.

### IL-13-induced regulation of unliganded and ligand-dependent GR phosphorylation and transactivation

**Figure 4:**
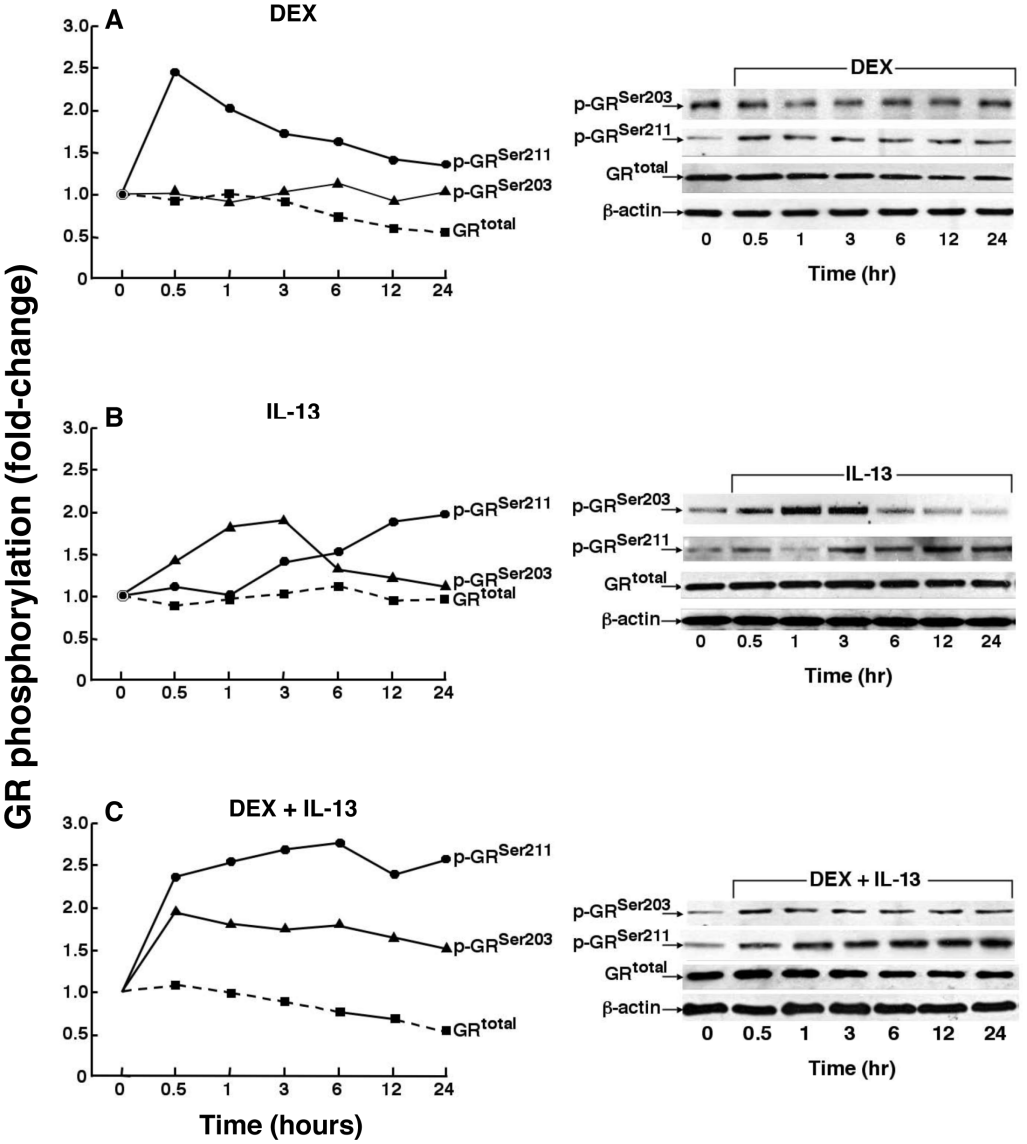
Comparison of induced temporal changes in site-specific GR phosphorylation in HASM cells treated with DEX or IL-13 alone and in combination. Representative immunoblots from one of three experiments depicting that, nder basal conditions, relative to p-GR^Ser211^, HASM cells exhibit distinctly increased p-GR^Ser203^ levels. Contrasting the acute transient increase in p-GR^Ser211^ elicited by DEX treatment alone (**A**), HASM cells treated with IL-13 alone exhibit an initial increase in p-GR^Ser203^ followed by a progressive increase in p-GR^Ser211^ (**B**), whereas combined treatment with DEX+IL-13 evokes sustained increases in p-GR^Ser203^ and p-GR^Ser211^ for up to 24 h (**C**). Note, GR^total^ levels are unaffected in HASM cells exposed to IL-13. By comparison, cells treated with DEX (alone or in combination with IL-13) exhibit progressive decreases in GR^total^ levels.

Whereas the intra-nuclear presence of p-GR^Ser211^ was shown to denote the hormone-activated state of GR, intra-cytoplasmic phosphorylation of GR^Ser203^ has also been demonstrated in the presence of GC ligand, and a potential interdependence of phosphorylation at these serine sites has been proposed [23]. To test whether HASM cells exhibit such interdependence in GR phosphorylation in the presence of ligand-dependent and -independent GR stimulation, anti-p-GR^Ser203^- and anti-p-GR^Ser211^-specific antibodies were used to initially determine the kinetics of GR phosphorylation at these serine sites in HASM cells that were treated for up to 24 h with either DEX or IL-13 alone and in combination. The representative immunoblots and corresponding densitometric analysis of immunoreactivity shown in Fig. 4A demonstrate that relatively little p-GR^Ser211^ was detected in the absence of ligand and that treatment with DEX elicited an early acute increase in p-GR^Ser211,^ wherein the level of immunoreactivity peaked at ∼2.5-fold above baseline at 0.5 h (mean = 2.97±0.64-fold in n = 4 experiments; p<0.05) and progressively decreased thereafter, but remained elevated above baseline at 24 h. By comparison, while distinctly present in the absence of ligand, GR^Ser203^ phosphorylation did not notably change following DEX administraion. Contrasting these responses to DEX, as shown in Fig. 4B, HASM cells treated with IL-13 alone exhibited an early progressive increase in p-GR^Ser203^ that peaked at 3 h (mean = 2.3±0.52-fold; p<0,05) and gradually declined thereafter, whereas p-GR^Ser211^ increased progressively after 1 h, with enhanced sustained phosphorylation at 24 h peaking at ∼2.0-fold (mean = 2.39±0.49-fold; p<0.05). By comparison, as shown in Fig. 4C, cells exposed to DEX+IL-13 displayed an early increase in p-GR^Ser203^ and, contrasting the transient response to DEX treatment alone, elevated p-GR^Ser211^ levels were sustained by 24 h (mean = 3.47±0.68-fold; p<0.05). In relation to these observations, it will be noted that total GR protein (GR^total^) expression was unaffected by IL-13 exposure alone and, while treatment with DEX, either alone or in combination with IL-13, had little effect initially, GR^total^ levels progressively decreased after ∼2 h to a nadir averaging 55.9±5.8% of baseline at 24 h in DEX±IL-13-treated cells. This reduction in GR expression in DEX-exposed cells, but not in unliganded cells treated with IL-13 alone, is consistent with the well documented observation that the GR undergoes down-regulation following ligand exposure, a finding reportedly attributed to enhanced GR mRNA and protein turnover in the continued presence of hormone [26], [27].

**Figure 5:**
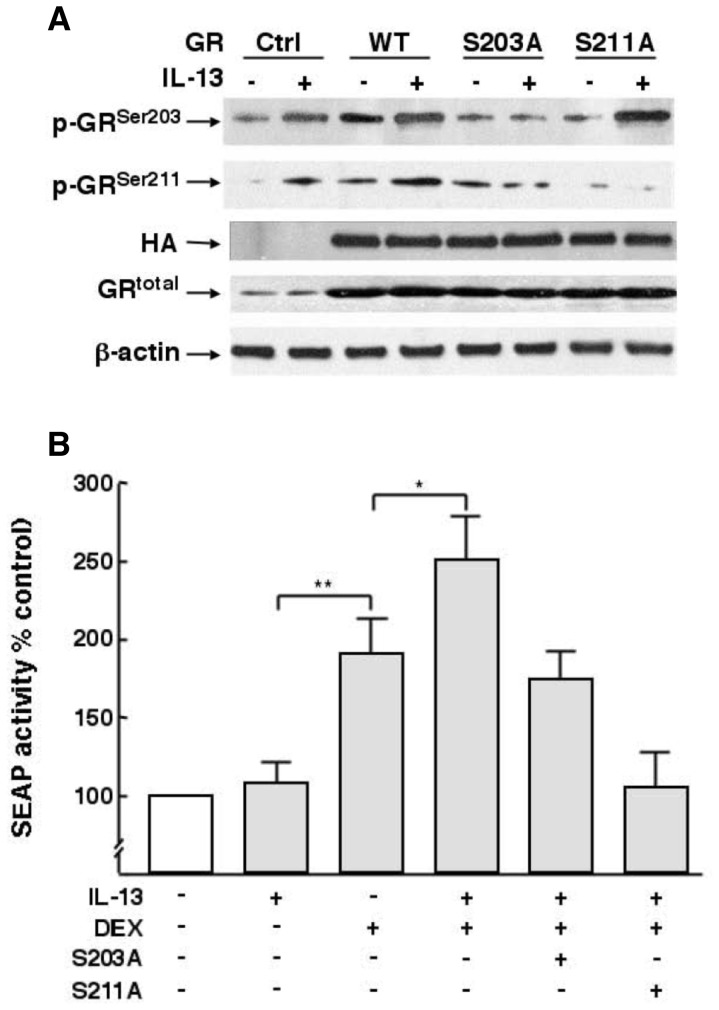
Interdependence of IL-13-induced GR^Ser203^ and GR^Ser211^ phosphorylation in HASM cells: Role in regulating GR tranactivation. (**A**) Immunoblots using anti-HA- and anti-GR phosphorylation-specific antibodies comparing the effects of IL-13 on GR^Ser203^ and p-GR^Ser211^ phosphorylation in HASM cells transfected with wild-type (WT) and either Ser^203^ or Ser^211^ phosphorylation-deficient mutant GR (S203A or S211A). Note: Under similar loading conditions, given by comparable levels of GR^total^ detected with anti-HA antibody, IL-13-induced increase in GR^Ser211^ phosphorylation is prevented in HASM cells transfected with the S203A mutant GR, whereas phosphorylation of GR^Ser203^ by IL-13 is unaffected in cells transfected with the S211A mutant construct (**B**) Comparison of effects of IL-13 on DEX-induced GR transactivation, detected using the SEAP reporter assay, in control HASM cells and cells transfected with either the S203A or S211A phosphorylation-deficient mutant GR. Note: Contrasting the lack of effect of IL-13 alone, SEAP activity is significantly increased in DEX-treated cells, and the response to DEX is significantly enhanced in the presence of IL-13. The latter stimulatory effect of IL-13 on DEX-induced GR transactivation is abrogated in cells transfected with the S203A mutant construct, and GR transactivation by DEX is prevented in cells transfected with the S211A mutant GR. Data represent mean ± SE values from 4–5 experiments (*p<0.05; **p<0.01).

To next examine whether the above IL-13-induced temporal changes in site-specific GR phosphorylation are causally related, the effects of IL-13 on peak GR^Ser203^ and GR^Ser211^ phosphorylation, determined at 3 and 24 h, respectively, were assessed in HASM cells that were transfected to overexpress hemagglutinin (HA)-tagged CMV promotor-driven wild-type human GR (pCMV-HA-hGR) and either of the alanine-substituted for serine site-specific mutant GRs, pCMV-HA-hGRS203A (S203A) or pCMV-HA-hGRS211A (S211A) (plasmids kindly provided by M. J. Garabedian; NYU, New York, NY). Immunoblotting using anti-HA- and anti-GR-specific antibodies demonstrated that, under similar loading conditions, as evidenced by the same level of GR^total^ detected with anti-HA antibody (Fig. 5A): 1) unlike in non-transfected (control) and wild-type (WT) GR-transfected cells wherein IL-13 elicited increases in both p-GR^Ser203^ and p-GR^Ser211^, minimal p-GR^Ser211^ was detected in IL-13-exposed S203A mutant transfected cells; and 2) by comparison, as in control and WT cells, IL-13-induced upregulation of p-GR^Ser203^ was distinctly present in cells transfected with S211A mutant construct. The observation that IL-13-induced GR^Ser211^ phosphorylation is suppressed in cells transfected with the S203A construct suggests that the above depicted initial phosphorylation of GR^Ser203 by^ IL-13 (Fig. 4B) is needed to enable subsequent GR phosphorylation at Ser^211^. Thus, these data are consistent with an earlier report implicating the presence of interdependence of GR phosphorylation at the serine 203 and 211 sites [23]. Of note, comparable experiments demonstrated that, unlike with IL-13, DEX-induced GR^Ser211^ phosphorylation, which is not associated with initial GR^Ser203^ phosphorylation (Fig. 4A), was unaffected by transfection with the S203A mutant (data not shown).

Given the above evidence of interdependence of GR phosphorylation at its serine 203 and 211 sites in IL-13-exposed cells, we reasoned that the sustained heightened GR^Ser211^ phosphorylation exhibited in cells treated with DEX+IL-13 (Fig. 4C) likely accounts for the increased DEX-induced GR:DNA binding activity detected in the presence of IL-13 (Fig. 3B). Accordingly, we next examined whether the latter effect of IL-13 confers enhanced hormone-stimulated GR transcriptional activity. As previously described (25), GR transactivation was assessed in HASM cells transfected with a promoter-reporter construct comprising a secretory alkaline phosphatase (SEAP) reporter driven by a GRE-dependent promoter (pGRE-SEAP). The transfected cells were treated with IL-13 (50 ng/ml) or DEX (1 µM) alone and in combination and, 24 h thereafter, the culture media was assayed by chemiluminescence for SEAP activity attributed to GR transactivation. As shown in Fig. 5B, contrasting the lack of effect of treatment with IL-13 alone, SEAP activity was significantly increased in HASM cells exposed to DEX, and this stimulatory effect of DEX was significantly enhanced in cells co-treated with IL-13, in concert with the above observed increased GR:DNA binding activity (Fig. 3B) associated with hyperphosphorylation of GR^Ser211^ in DEX+IL-13-treated cells (Figs. 2C and 4). This IL-13-induced heightened hormone-stimulated GR transcriptional activation was prevented in IL-13-exposed that were transfected with the S203A mutant, wherein SEAP activity was similar to that detected in cells exposed to DEX alone. Not surprisingly, given the known critical role of Ser211 phosphorylation in mediating hormone-stimulated GR transactivation, induction of SEAP activity by DEX was suppressed in HASM cells transfected with the S211A mutant construct (Fig. 5B).

### MAPK activation mediates effects of IL-13 on DEX-induced GR transcriptional activity

**Figure 6:**
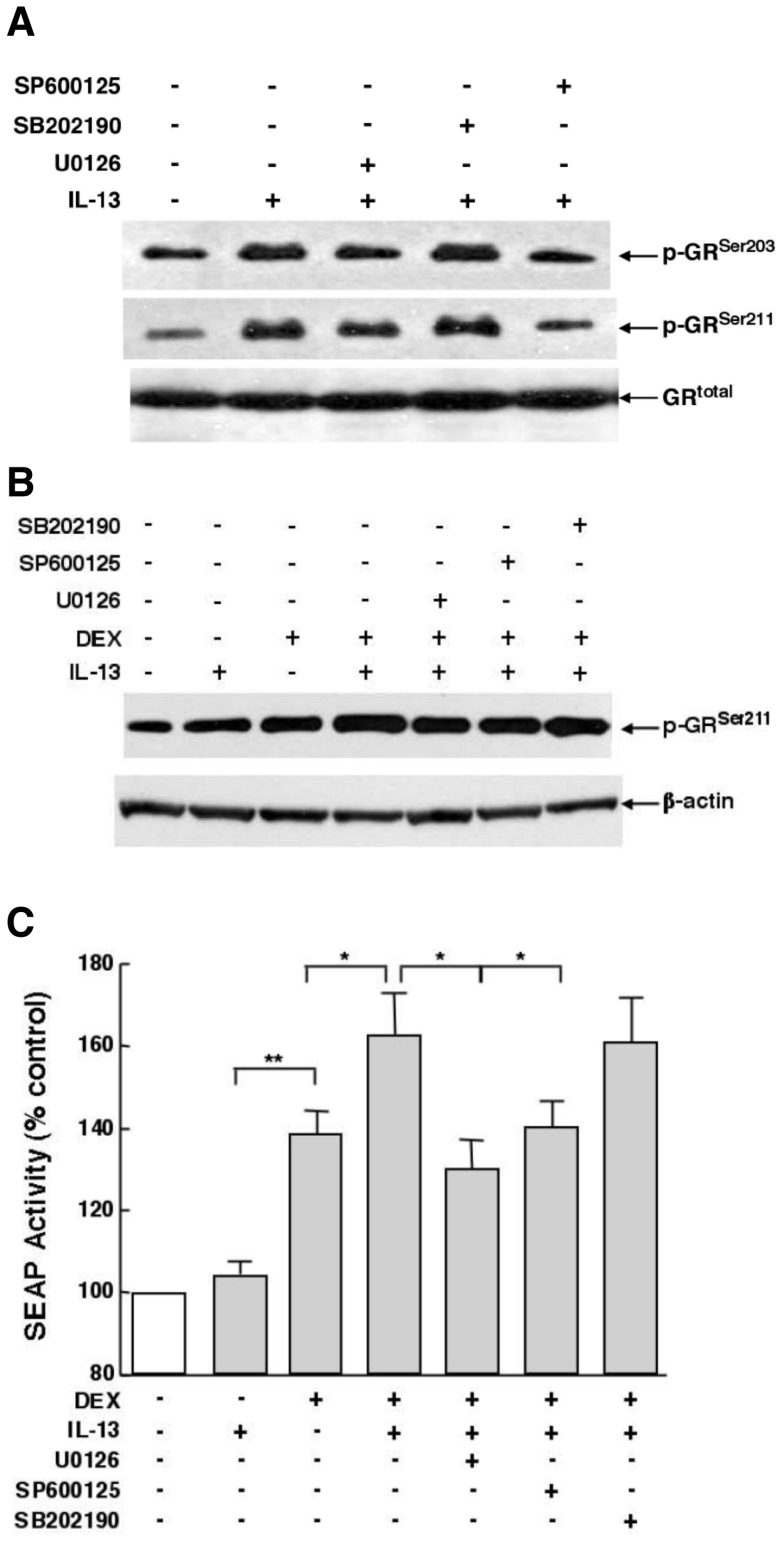
GR phosphorylation by IL-13 and its induction of heightened DEX-stimulated GR^Ser211^ phosphorylation and transactivation are suppressed by inhibition of ERK1/2 and JNK signaling. (**A**) Representative immunoblots from one of three experiments demonstrating that IL-13-induced maximal GR^Ser203^ and GR^Ser211^ phosphorylation elicited at 3 and 12 h, respectively, are suppressed in HASM cells pretreated with either the ERK1/2 or JNK inhibitors, U0125 and SP600125, respectively, whereas pretreatment with the p38 MAPK inhiobitor, SB202190, has no appreciable effect. Comarably, heightened DEX-stimulated GR^Ser211^ phosphorylation (B) and transactivation of SEAP activity (C) exhibited in IL-13-exposed HASM cells are suppressed by pretreatment with the ERK1/2 and JNK inhibitors, whereas the p38 MAPK inhiobitor has no effect. Data are mean ± SE values from 4 experiments (*p<0.05; **p<0.01).

The pro-asthmatic effects of IL-13 in HASM cells were previously shown to be largely mediated by MAPK signaling, specifically involving IL-13-induced acute ERK1/2 and JNK activation [25], [28], [29]. Given this evidence, together with that demonstrating that MAPK activation regulates GR phosphorylation in various cell types [13]–[15], the potential role of MAPK signaling in regulating IL-13-induced changes in GR phosphorylation and its transcriptional activity in HASM cells was examined both in the absence and presence of hormone stimulation. Initial studies investigating the effects of specific MAPK inhibitors on IL-13-induced phosphorylation of GR^Ser203^ and GR^Ser211^ demonstrated that the peak increases in both p-GR^Ser203^ and p-GR^Ser211^ detected at 3 and 12 hr following IL-13 administration, respectively (Fig. 4), were inhibited in HASM cells pretreated with either the MEK-ERK1/2 inhibitor, U0126 (5 µM), or the JNK inhibitor, SP600125 (10 µM), whereas the p38 MAPK inhibitor, SB202190 (10 µM), had no inhibitory effect (Fig. 6A). Subsequent examination of the effects of these inhibitors on GR^Ser211^ phosphorylation in cells exposed to IL-13 in the absence and presence of hormone stimulation demonstrated that, relative to the increases in p-GR^Ser211^ phosphorylation elicited in cells treated with either IL-13 (50 ng/ml×24 h) or DEX (1 µM×1 h) alone, the hyperphosphorylation of GR^Ser211^ detected in DEX+IL-13-exposed cells was abrogated by pretreatment with either the ERK1/2 or JNK inhibitor, whereas the p38 MAPK inhibitor had no appreciable effect (Fig. 6B). Similar results to those exemplified in Figs. 6A and 6B were obtained in additional experiments, as demonstrated by quantitative densitometric analysis of the fold-changes in p-GR^Ser203^ and p-GR^Ser211^ expression detected under the same treatment conditions (Fig. S2). Moreover, these results were comparable to those obtained when using the SEAP reporter assay to assess the effects of these treatment conditions on GR transcriptional activity (Fig. 6C). Accordingly, in concert with its aforementioned lack of effect on GR:DNA binding activity (Fig. 3A), IL-13 treatment alone had no significant effect on SEAP activity. By comparison, as expected, SEAP activity was significantly increased in cells exposed to DEX; and the stimulatory effect of DEX was significantly enhanced in cells co-treated with IL-13, in accordance with both the corresponding hyperphosphorylation of GR^Ser211^ (Fig. 6B) and heightened GR:DNA binding activity detected in DEX+IL-13-exposed cells (Fig. 3B). The augmented hormone-stimulated transcriptional activity was abrogated by pretreating DEX+IL-13-exposed cells with either the ERK1/2 or JNK inhibitor, whereas the p38 MAPK inhibitor had no effect. Collectively, these data are consistent with the notion that, while having no effect alone on GR:DNA binding activity, IL-13 enhances hormone-stimulated GR transcriptional activity due to hyperphosphorylation of GR^Ser211^ that is attributed to IL-13-induced activation of the GR, associated with MAPK-dependent GR^Ser203^ phosphorylation, which upregulates ligand-stimulated GR^Ser211^ phosphorylation.

**Figure 7:**
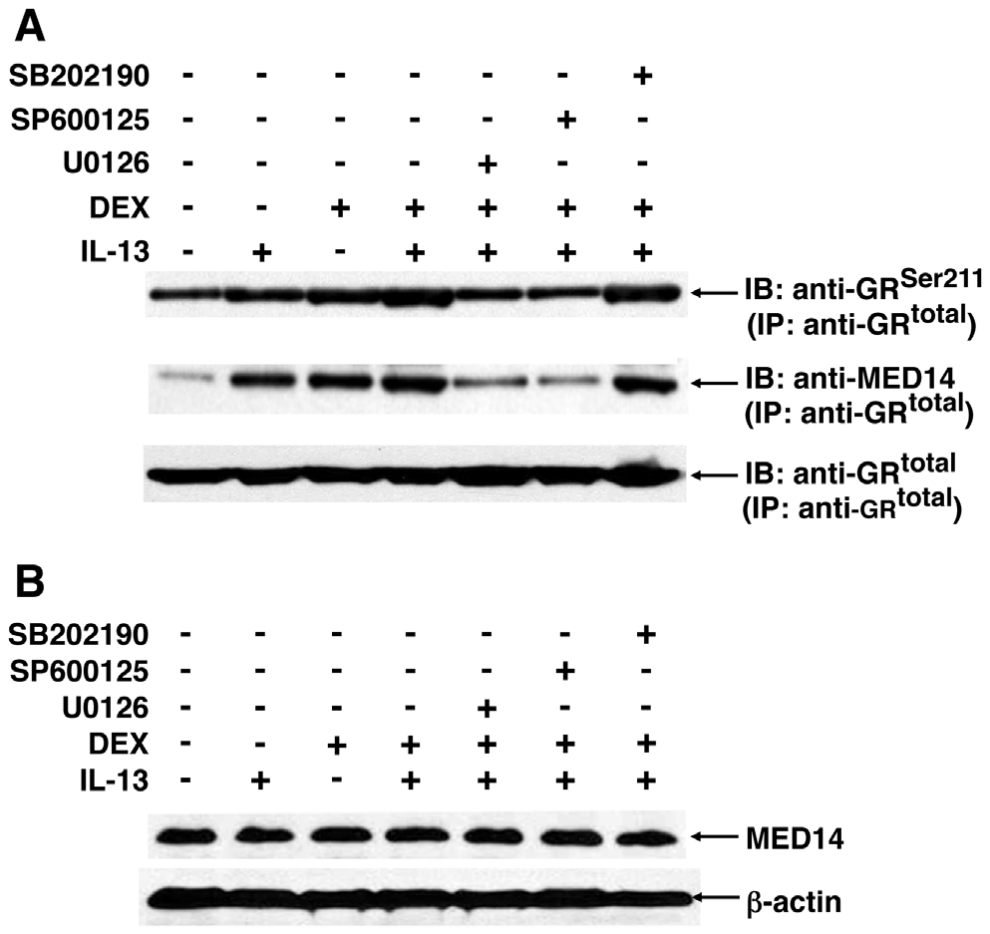
Enhanced DEX-induced co-localization of p-GR^Ser211^ and the transcriptional co-factor, MED14, in IL-13-exposed HASM cells is prevented by inhibition of ERK1/2 and JNK signaling. (**A**) Representative co-immunoprecipitation study demonstrating increased co-localization of both phosphorylated GR^Ser211^ and MED14, detected by immunoblotting (IB), with immunoprecipitated (IP) GR in nuclear extracts from HASM cells exposed to either IL-13 or DEX alone. The latter co-localiztion is further enhanced in IL-13+DEX-treated cells, and is largely suppressed by pretreating the cells with either the ERK1/2 or JNK inhibitor, whereas the p38 MAPK inhibitor has no effect. (**B**) Note: The above effects elicited under the different treatment conditions are detected in the absence of any corresponding change in total MED14 protein expression.

The above notion was further substantiated in co-immunoprecipitation experiments wherein the effects of the MAPK inhibitors on IL-13-induced changes in hormone-stimulated recruitment of MED14, the transcriptional co-factor associated with GR hyperphosphorylation coupled to heightened transactivation [13], [18], were examined in DEX-exposed HASM cells. The immunoblots (IB) in Fig. 7A demonstrate that, relative to unstimulated cells, increased co-localization of both phosphorylated GR^Ser211^ and MED14 with immunoprecipitated (IP) GR was detected in nuclear extracts from HASM cells exposed to either IL-13 or DEX alone, and this co-localiztion was further enhanced in IL-13+DEX-treated cells. The latter heightened co-localization was largely abrogated in IL-13+DEX exposed cells that were pretreated with either U0126 or SP600125, implicating regulatory roles for ERK1/2 and JNK activation, respectively, whereas the p38 MAPK inhibitor had no effect. Of note, the above observed effects were detected in the absence of any corresponding changes in total MED14 protein expression under the same treatment conditions (Fig. 7B). Thus, together with the above observations, these data support the concept that IL-13-regulated heightened phosphorylation of the hormone-stimulated GR, coupled to recruitment of MED14 to the hyperphosphorylated GR, is reponsible for the increased DEX-stimulated GR transcriptional actvity exhibited in the presence of the cytokine.

## Discussion

The bronchoprotective action of GCs in asthma therapy is not only due to their anti-inflammatory properties but also reflects their ability to directly act on ASM by inhibiting its contractility, increasing its relaxation, inhibiting ASM cell proliferation, preventing the release of various proinflammatory cytokines and chemokines from the pro-asthmatic sensitized ASM, and additional actions [22], [30], [31]. As in other tissues, an important determinant of ASM responsiveness to endogenous and a variety of synthetic GCs is its ability to regulate pre-receptor GC bioavailability by modulating the activities of the GC-activating and -inactivating isozymes, 11ß-HSD1 and HSD2, respectively [25], [32]. Accordingly, exposure of HASM cells to proinflammatory cytokines (e.g., IL-13, IL-1β/TNFα, etc) was shown to upregulate 11ß-HSD1 and inhibit 11ß-HSD2 expression, thereby allowing for increased conversion of cortisone into its bioactive derivative, cortisol [22], [25], [32], [33], which serves to homeostatically suppress the opposing pro-asthmatic effects of the cytokine on airway contractility [25], [32]. The present study sought to determine whether the homeostatic response of HASM to proinflammatory cytokine exposure also includes changes in GR signaling by exploring unliganded and ligand-dependent GR activation in HASM cells stimulated by the key pro-asthmatic cytokine, IL-13. The results are the first to demonstrate that: 1) IL-13-exposed HASM cells exhibit ligand-independent activation of the GR that involves its nuclear translocation coupled to MAPK-regulated sequential phosphorylation of GR at its serine 203 and 211 sites; and 2) this cytokine-driven unliganded GR response confers increased GC hormone-stimulated GR^Ser211^ phosphorylation and DNA binding activity that, in association with enhanced recruitment of the co-factor, MED14, to the hyperphosphorylated GR, allows for heightened GR transactivation. Together, these novel findings highlight a heretofore unidentified homeostatic cytokine-driven GR signaling mechanism in HASM that involves ligand-independent GR activation which enables increased responsiveness to GC hormone treatment. This intrinsic homeostatic mechanism may serve an important role by enhancing the efficacy of GC therapy in mitigating the airway asthmatic response.

A number of previous studies have demonstrated the presence of ligand-independent activation of steroid receptors in different cell types, including the estrogen [34], [35], progesterone [36], and androgen [37] receptors, and there is now emerging evidence that also implicates ligand-independent activation of the GR. In this regard, stimulation of lung fibroblasts and vascular smooth muscle cells with β2-adrenoceptor agonists was shown to evoke ligand-independent GR activation [38], and unliganded GR activation was also demonstrated in ursodeoxycholic bile acid-treated hamster ovary cells [39], LβT2 and COS-1 cells stimulated with gonadotropin-releasing hormone (GnRH) [40], and TNFα treatment of a human endocervical epithelial cell line. End1/E6E7, and COS-1 cells [41]. In general agreement with this recent evidence, our present observations demonstrate that proinflammatory cytokine-exposed HASM cells exhibit acute nuclear translocation coupled to phosphorylation of the unliganded GR. These data raise certain noteworthy issues. First, in demonstrating that GR translocation was elicited in HASM cells that were exposed to either the Th2 cytokine, IL-13, or the pleitropic cytokines, IL-1β and TNFα, our results imply that the induced unliganded GR response is not cytokine-specific, as least with respect to the latter proinflammatory cytokines (discussed below). Secondly, to the extent that the magnitude of intra-nuclear immunofluorescence staining of GR detected in HASM cells treated with IL-13+DEX was roughly comparable to the additive effects of IL-13 and DEX alone (Fig. 1), these data demonstrate that the responses to ligand-dependent and -independent GR activation are not mutually exclusive but, rather, appear synergistic in nature. This notion was further substantiated by the observation that intra-nuclear accumulation of phosphorylated GR^Ser211^, which denotes the activated state of the receptor, was distinctly increased in IL-13+hormone (cortisone or DEX)-treated HASM cells relative to cells exposed to hormone alone (Fig. 2). In this regard, it should be noted that the increased phosphorylated GR^Ser211^ detected in IL-13+hormone-stimulated cells denoted the presence of heightened GR activation as this response was inhibited by pretreatment with RU486, a well characterized GR antagonist that has a high affinity for the receptor [42].

Post-translational modification of GR is known to regulate various aspects of GR signaling including its nuclear translocation, co-factor recruitment, DNA binding activity, and transactivation or transrepression functions. In addition to Ser^211^ phosphorylation, key sites for such covalent modification of the human GR in the AF1 region include residues Ser^203^ and Ser^226^ [13], [16], [18]. Whereas Ser^226^ phosphorylation has been associated with attenuated GR signaling and transcriptional repression [18], [20], [23], [41], [43], Ser^203^ phosphorylation is preferentially retained in the cytoplasm and has been implicated in regulating hormone-stimulated Ser^211^ phosphorylation, resulting in increased GR transcriptional activity [18], [23]. The latter interdependence of GR phosphorylation was also demonstrated herein when examining the effects of IL-13 on unliganded and DEX-stimulated GR phosphorylation, GR:DNA binding activity, and transcriptional activation. In this connection, the results demonstrated that IL-13 alone elicits sequential GR phosphorylation, initially at Ser^203^ followed by progressive and sustained Ser^211^ phosphorylation (Fig. 4B), and that these temporal patterns of site-specific GR phosphorylation are causally related, as evidenced by abrogation of IL-13-induced Ser^211^ phosphorylation in HASM cells that are transfected with the S203A mutant construct (Fig. 5A). While this observation concurs with earlier evidence demonstrating an interdependece of Ser^203^ and Ser^211^ phosphorylation in other cell types (23), it should be noted that our observed unliganded p-GR^Ser203^ -dependent phosphorylation of GR^Ser211^ in IL-13-exposed HASM cells was not accompanied by changes in either GR:DNA binding activity or GR transactivation. Rather, exposure to IL-13 was found to enhance hormone-stimulated GR signaling, as demonstrated by sustained augmented GR^Ser211^ hyperphosphorylation, increased GR:DNA binding activity, and heightened GR transactivation in DEX+IL-13-exposed HASM cells, as compared to the corresponding responses exhibited in cells treated with DEX alone. Moreover, in concert with the observed suppression of IL-13-induced Ser^211^ phosphorylation in cells transfected with the S203A mutant (Fig. 5A), the heightened GR transactivation detected in IL-13+DEX-exposed cells was also curtailed in cells transfected with the phosphorylation-deficient S203A mutant construct (Fig. 5B). Thus, given the different temporal patterns of serine site-specific GR phosphorylation, together with the lack of induced GR:DNA binding activity and GR transactivation, the mechanism of IL-13-stimulated unliganded GR activation appears to be qualitatively different from that elicited by hormone exposure alone in HASM cells. Nevertheless, although arguably different, the mehanisms regulating the unliganded and ligand-induced GR responses act synergistically to heighten the net hormone-stimulated GR transcriptional response.

When evaluated in light of recent reports, the present results raise certain relevant considerations regarding the phenomenon of unliganged GR signaling. In this respect, whereas our observations in IL-13-exposed HASM cells are consistent with the finding of acute nuclear translocation of the non-ligand-stimulated GR in other cell types [38], [39], [41], we found no evidence that this early IL-13-stimulated signaling event is associated with altered GR:DNA binding activity or GR-regulated transactivation. This contrasts with the reported observations that unliganded GR activation is accompanied by induced GR:DNA binding and transactivation in various cell types including β2-adrenoceptor-stimulated lung fibroblasts and vascular smooth muscle cells [38], ursodeoxycholic-treated hamster ovary cells [39], and LβT2 and COS-1 cells stimulated with GnRH [40]. While this disparity is not readily explained, apart from differences potentially related to cell type, GRα isoform-specific activation, post-translational GR modifications, and other factors, consideration must also be given to the specific experimental conditions used to elicit unliganded GR activation. This issue is exemplified when comparing our present observations involving proinflammatory cytokine exposure in HASM cells to those recently reported using TNFα in End1/E6E7 and COS-1 cells, wherein treatment with the cytokine was found to induce phosphorylation of the unliganded GR at Ser^226^ (and not at Ser^211^) which, along with recruitment of the GR co-factor, GRIP-1, was required for ligand-independent *repression* of IL-6 expression in response to TNFα [41]. By comparison, our data demonstrated that IL-13 elicits ligand-independent MAPK-mediated GR^Ser203^ phosphorylation that evokes Ser^211^ phosphorylation of the unliganded GR, and that this covalent modification is heightened in the presence of hormone stimulation in association with increased recruitment of MED14 to the hyperphosporylated GR, and consequent increased hormone-stimulated GR:DNA binding activity and transactivation. Of note, the latter mechanistic scenario fundamentally agrees with that proposed in an earlier study demonstrating that steroidogenic factor 1 (SF-1), a nuclear receptor that regulates many peptide hormone-induced genes, also exhibits ligand-independent MAPK-mediated phosphorylation of SF-1 at its Ser^203^ residue in the AF1 region, which facilitates co-factor recruitment that then confers coordinated regulation of multiple SF-1 target genes in response to hormone stimulation [44].

In view of earlier studies demonstrating that the pro-asthmatic effects of IL-13 in HASM cells are largely attributed to MAPK activation [24], [28], [29], and given our recent finding that acute activation of the MAPKs, ERK1/2 and JNK, mediates IL-13-induced upregulation of the GC-activating enzyme, 11β-HSD1 in HASM cells [24], the potential role of MAPK activation in regulating IL-13-induced GR signaling was examined herein. Our results demonstrated that inhibition of ERK1/2 and JNK activation suppressed the stimulatory effects of IL-13 on GR signaling, including the early and subsequent IL-13-induced phosphorylation of GR^Ser203^ and GR^Ser211^, respectively^,^ as well as the hyperphosphorylation of GR^Ser211^ and heightened GR transactivation accompanying increased recruitment of MED14 to the GR in DEX+IL-13-exposed cells. Taken together, these data are consistent with the important role previously attributed to MAPK activation (notably ERK1/2 and JNK) in regulating hormone-stimulated GR phosphorylation and its transcriptional response in other cell types [13], [18], [19], [45], [46], as well as unliganded GR phosphorylation by GnRH in the LβT2 cell line [40]. This evidence notwithstanding, it remains to be established whether our observations additionally reflect, at least in part, the contributions of other kinases that are also known to regulate human GR phosphorylation, including cyclin-dependent kinases and glycogen synthase kinase-3 [13].

The nature of the interaction between proinflammatory cytokines and GR signaling is complex and often viewed as mutually antagonistic, with studies reporting attenuated GR function in isolated peripheral blood mononuclear cells (PBMCs) exposed to proinflammatory cytokines and in PBMCs isolated from steroid-resistant asthmatic individuals [47]–[50]. Specifically, regarding the action of IL-13, it was previously reported that the suppressive effect of hydrocortisone on LPS-induced IL-6 production is significantly attenuated in IL-13-treated human monocytes, and that this suppressive effect is attributed to IL-13-induced reduction in GR binding affinity [50]. Conversely, other studies have reported an increase in GR expression under different proinflammatory conditions and following treatment of different cell lines, including airway epithelial cells, with proinflammatory cytokines [8]–[11]. While these seemingly discordant previous findings are not easily reconciled, they may be reflective (at least partially) of the effects of specific cytokine treatments on hormone-stimulated GR signaling. This notion is evidenced when considering the findings in recent studies that examined the effects of different cytokine treatments on GC responses in HASM cells. Accordingly, whereas treatment of HASM cells with the combination of TNFα and IFNγ was recently shown to inhibit GR^Ser211^ phosphorylation by the GC ligand, fluticasone, presumably due to increased phosphatase-specific activity [51], it was previously reported that, unlike with TNFα and IFNγ, suppression of GC action in HASM cells was not seen with the treatment combinations of TNFα and either IL-1β or IL-13 [52]. Although the latter studies did not examine the unliganded effects of cytokine stimulation alone, given that our present observations demonstrate that IL-13 and combined treatment with TNFα+IL-1β elicit cooperative ligand-independent and -dependent stimulatory effects on GR activation, it appears that, even within the same cell type, the effects of cytokine stimulation on GC responsiveness are selective to certain cytokines or their combination. This issue emphasizes the need for future in depth studies to address the interactions between individual and combined cytokine treatments and GR signaling.

**Figure 8:**
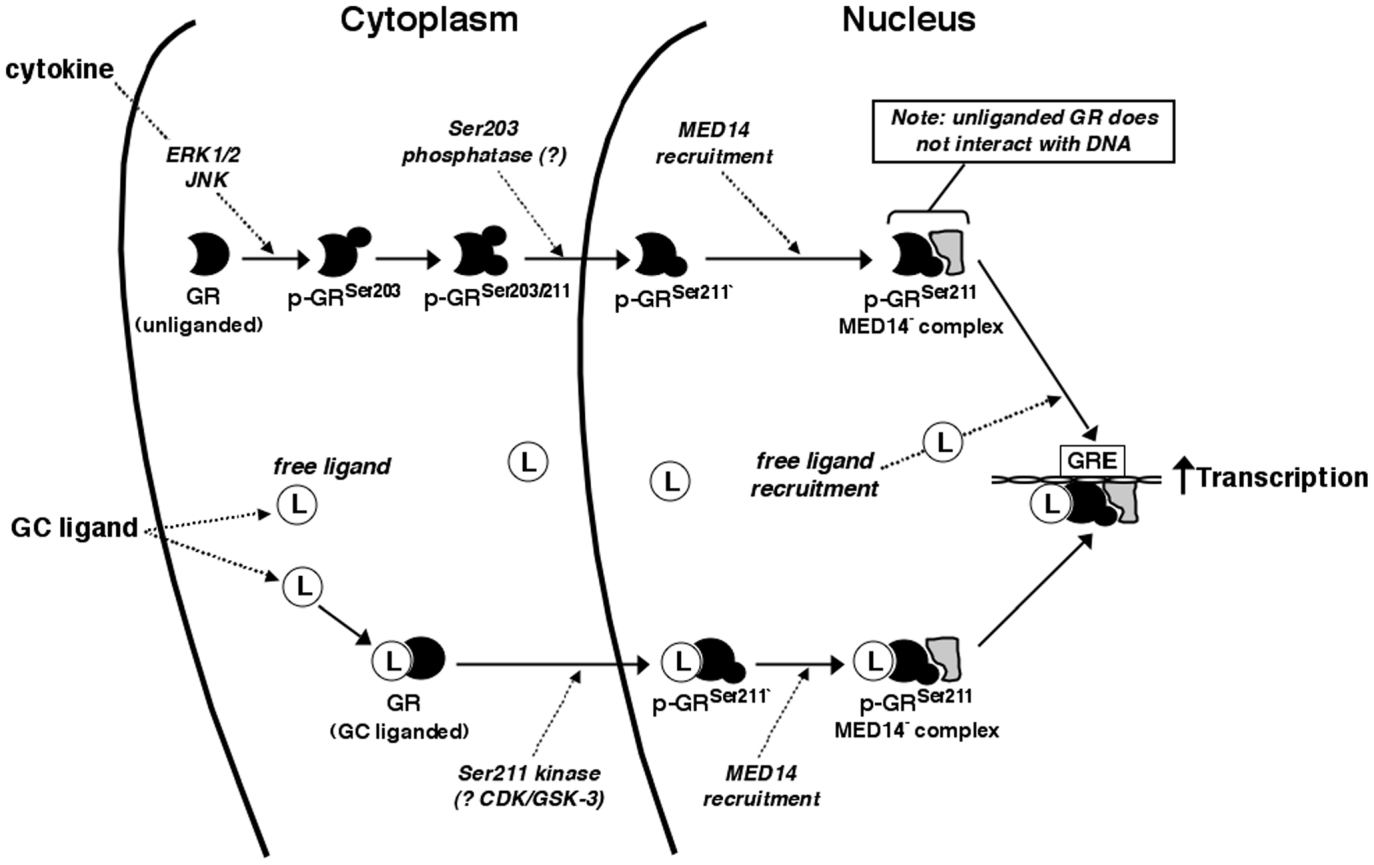
Schematic representation of proposed homeostatic mechanism involving cytokine-stimulated ligand-independent GR activation and its induction of heightened ligand-stimulated GR signaling and transactivation. Cytokine (IL-13) exposure acutely elicits ERK1/2- and JNK-mediated GR^Ser203^ phosphorylation that leads to p-GR^Ser203^-dependent GR^Ser211^ phosphorylation. The dual GR^Ser203/211^ phosphorylated state is transient, as Ser203 becomes dephosphorylated (unidentified phosphatase (13)) and p-GR^Ser211^ translocates to the nucleus where it associates with the transcriptional co-factor, MED14. In the absence of hormone, the unliganded p-GR^Ser211^-MED14 complex is incapable of binding to DNA. With hormone exposure, ligand entering the cell can translocate to the nucleus both as free and GR-bound ligand, the latter leading to GR^Ser211^ phosphorylation potentially via the kinase activities of cyclin-dependent kinase (CDK) or glycogen synthase kinase-3 (GSK-3). Note: relative to ligand treatment alone, combined cytokine+ligand exposure elicits increased intra-nuclear levels of p-GR^Ser211^ coupled to MED14, leading to heightened GR transcriptional activity upon recruitment of free ligand to the cytokine-stimulated unliganded p-GR^Ser211^-MED14 complex.

In conclusion, this study examined the effects and mechanisms of action of pro-asthmatic cytokine exposure in regulating both ligand-independent and -dependent GR signaling in HASM cells. The data are the first to demonstrate that: 1) HASM cells exposed to IL-13 exhibit ligand-independent GR activation that involves its nuclear translocation and MAPK-mediated sequential serine site-specific phosphorylation, coupled to recruitment of the MED14 co-factor to the unliganded phosphorylated GR; and 2) this unliganded GR activation enables heightened hormone-stimulated GR signaling in the cytokine-exposed HASM cells wherein increased GR translocation and GR^Ser211^ phosphorylation confers emhanced hormone-stimulated GR:DNA binding, recruitment of MED14, and transactivation. Thus, these data highlight a heretofore unidentified pro-asthmatic cytokine-driven homeostatic GR siganling mechanism in HASM cells that serves to heighten GC hormone responsiveness, as schematically depicted in Fig. 8. This new evidence, when considered in light of the challenge faced in treating a significant number of severely asthmatic individuals that are insensitive to GC therapy, raises the consideration that this GC insensitivity may be related, at least in part, to perturbations in the herein-described homeostatic cytokine-driven GR signaling mechanism. This possibility warrents systematic investigation since, if confirmed, it may lead to the development of future interventions targeted at such perturbations in GR signaling, thereby potentially enhancing the efficacy of GC treatment of asthma.

## Supporting Information

Figure S1 **Separate and combined effects of dexamethasone (DEX) and IL-β/TNFα on nuclear translocation of GR in HASM cells.** GR localization detected by immunofluorescence staining demonstrates that, relative to diffuse cytoplasmic distribution of GR in vehicle-exposed (control) HASM cells, treatment for 30 min with either DEX or IL-β/TNFα alone elicits increased intra-nuclear localization of GR. which appears further enhanced in cells co-treated with DEX ± IL-β/TNFα.
(TIF)

Figure S2 **Densitometric analysis of immunoblots depicting that GR phosphorylation by IL-13 and its induction of heightened DEX-stimulated GR^Ser211^ phosphorylation are suppressed by inhibition of ERK1/2 and JNK signaling.** (**A**) IL-13-induced increases in maximal levels of p-GR^Ser203^ and p-GR^Ser211^ detected at 3 and 12 h, respectively, expressed as fold-changes from baseline, are suppressed in HASM cells pretreated with either U0125 or SP600125, whereas pretreatment with SB202190 has no significant effect. (**B**) Similarly, increased levels of DEX-stimulated p-GR^Ser211^ in IL-13-exposed HASM cells are suppressed by pretreatment with the ERK1/2 and JNK inhibitors, whereas the p38 MAPK inhibitor has no effect. Data are mean ± SE values from n = 4 experiments under each treatment condition (*p<0.05).
(TIF)
