# Supplementary figures and images for: Pro-Asthmatic Cytokines Regulate Unliganded and Ligand-Dependent Glucocorticoid Receptor Signaling in Airway Smooth Muscle
Source: PLoS One. 2013 Apr 4;8(4):e60452. doi: 10.1371/journal.pone.0060452 (PMC3617099; doi:10.1371/journal.pone.0060452)

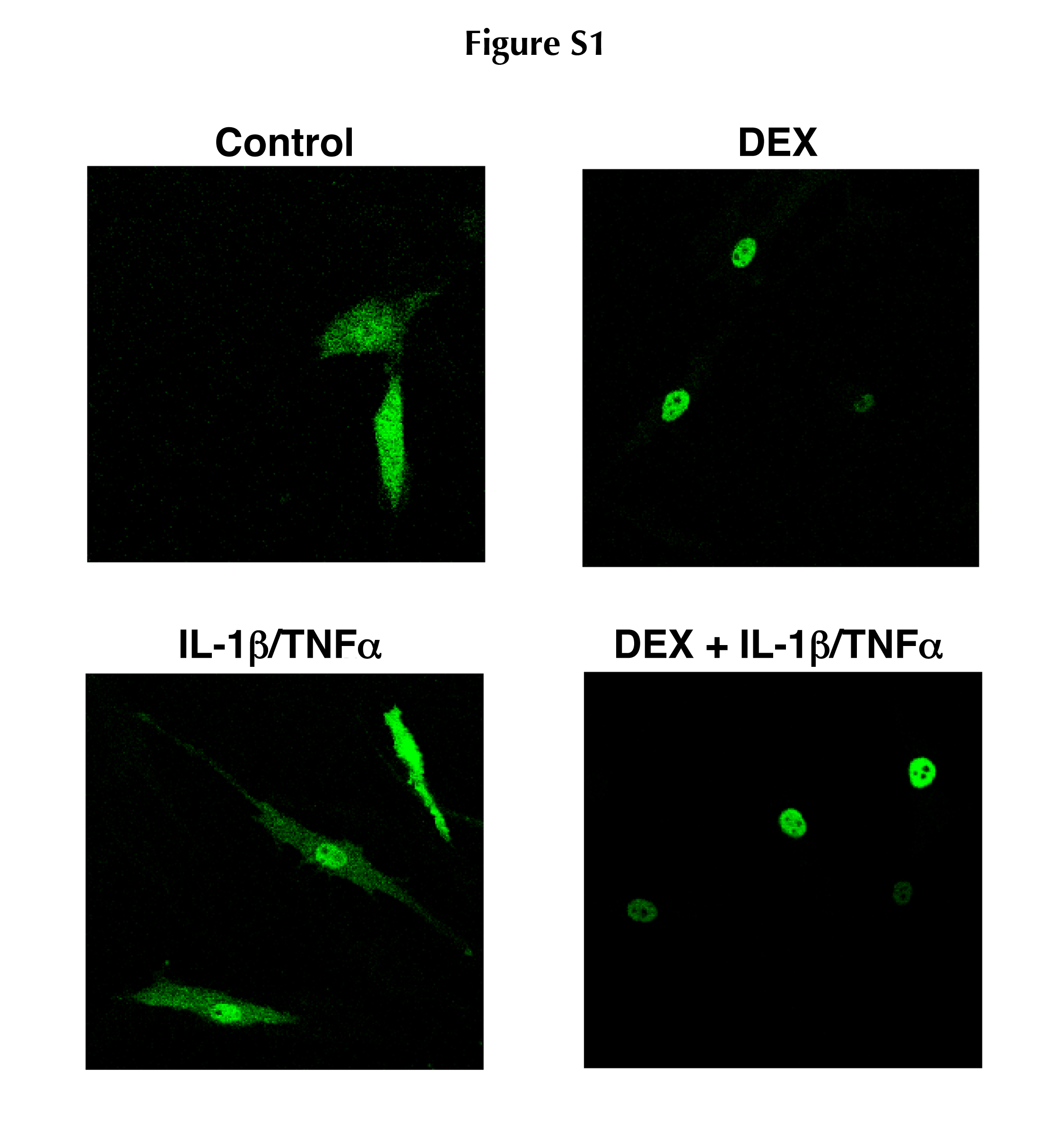

Supplement: Figure S1 — Separate and combined effects of dexamethasone (DEX) and IL-β/TNFα on nuclear translocation of GR in HASM cells. GR localization detected by immunofluorescence staining demonstrates that, relative to diffuse cytoplasmic distribution of GR in vehicle-exposed (control) HASM cells, treatment for 30 min with either DEX or IL-β/TNFα alone elicits increased intra-nuclear localization of GR. which appears further enhanced in cells co-treated with DEX ± IL-β/TNFα. (TIF) [file pone.0060452.s001.tif]

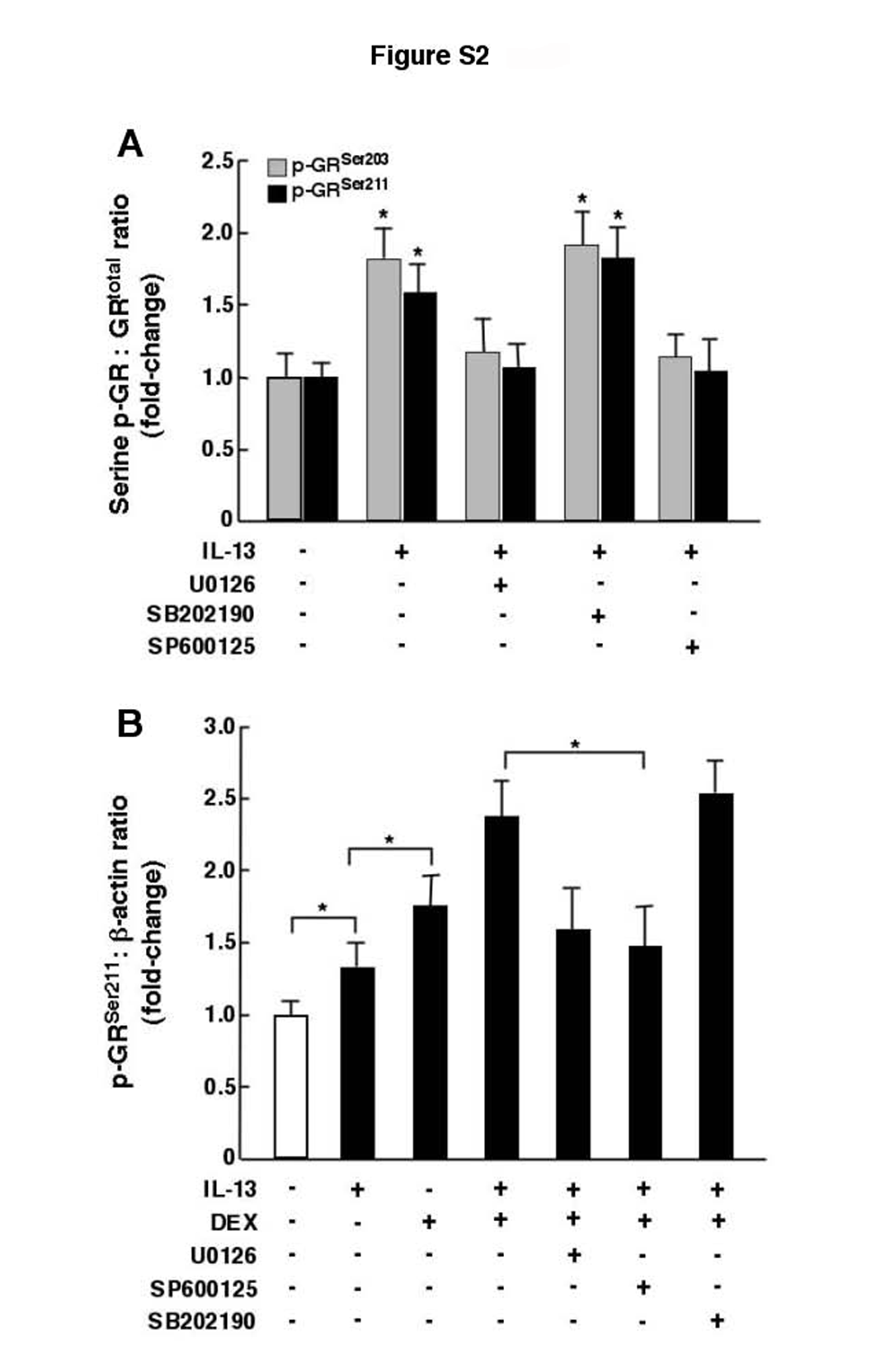

Supplement: Figure S2 — Densitometric analysis of immunoblots depicting that GR phosphorylation by IL-13 and its induction of heightened DEX-stimulated GRSer211 phosphorylation are suppressed by inhibition of ERK1/2 and JNK signaling. (A) IL-13-induced increases in maximal levels of p-GRSer203 and p-GRSer211 detected at 3 and 12 h, respectively, expressed as fold-changes from baseline, are suppressed in HASM cells pretreated with either U0125 or SP600125, whereas pretreatment with SB202190 has no significant effect. (B) Similarly, increased levels of DEX-stimulated p-GRSer211 in IL-13-exposed HASM cells are suppressed by pretreatment with the ERK1/2 and JNK inhibitors, whereas the p38 MAPK inhibitor has no effect. Data are mean ± SE values from n = 4 experiments under each treatment condition (*p<0.05). (TIF) [file pone.0060452.s002.tif]
